# *SDR3.1* negatively regulates seed dormancy in Poaceae crops

**DOI:** 10.1111/tpj.71141

**Published:** 2026-10-05

**Authors:** Naihui Guo, Ruihu An, Mengxue Xu, Yuanyuan Li, Zongliang Ren, Jun Jiang, Bonian Cai, Xinzhu Huang, Shikai Hu, Gaoneng Shao, Guiai Jiao, Lihong Xie, Ling Wang, Fengli Zhao, Yujun Zhu, Shaoqing Tang, Zhonghua Sheng, Peisong Hu

**Affiliations:** ^1^ State Key Laboratory of Rice Biological Breeding/Key Laboratory of Rice Biology and Breeding, Ministry of Agriculture/China National Rice Improvement Centre/Jiangxi Early‐season Rice Research Center/ China National Rice Research Institute Hangzhou P. R. China; ^2^ Zhejiang top Yunnong Technology Co., Ltd Hangzhou P. R. China

**Keywords:** rice, *SDR3.1*, *Sdr4*, seed dormancy, preharvest sprouting, Poaceae crops

## Abstract

Elucidating the molecular mechanisms that govern seed dormancy is important for developing crops with improved resistance to preharvest sprouting (PHS). A series of seed dormancy genes have been cloned, but the connections among different seed dormancy genes remain poorly understood. Here, we have revealed the relationship between two important dormancy quantitative trait loci (QTLs): *SDR3.1* and *Sdr4*. SDR3.1 formed complexes with ABI3 and ABI5 and suppressed their activation of *Sdr4* transcription. Haplotype analysis showed that the germination rate of *SDR3.1* L‐type and *Sdr4* n‐ or K‐type combinations was low, and the parents of modern core breeding do not contain l‐type. Analysis of transgenic plants confirmed that orthologs of SDR3.1 have conserved functions in controlling seed dormancy in rice (*Oryza sativa* L.), foxtail millet (*Setaria italica* (L.) *P. Beauv*.), and maize (*Zea mays*), but they have different molecular regulatory mechanisms. This study uncovers the OsSDR3.1‐ABI3/ABI5‐Sdr4 regulatory module conferring improved PHS resistance, while establishing an effective approach to characterize core functional genes for important agronomic traits across Poaceae species with rice as a model.

## Significance statement

For staple crops in the Poaceae family, such as wheat, barley, and maize, yield losses occur due to preharvest sprouting (PHS), in which seeds germinate on the mother plant directly before harvest when they encounter high temperature and humidity. This study uncovers the OsSDR3.1‐ABI3/ABI5‐Sdr4 regulatory module conferring improved PHS resistance, while establishing an effective approach to characterize core functional genes for important agronomic traits across Poaceae species with rice as a model.

## INTRODUCTION

Improvements in crop yield are getting smaller each year. Consequently, with the decreasing availability of arable land and the outbreak of extreme climate, the goal of ending global hunger by 2030 is becoming increasingly challenging (Hodson, 2017). As such, reducing yield losses is an important goal of current agricultural research. For staple crops in the Poaceae family, such as wheat, barley, and maize, yield losses occur due to preharvest sprouting (PHS), in which seeds germinate on the mother plant directly before harvest when they encounter high temperature and humidity (Xu et al., 2019). In addition, PHS can reduce seed quality and the lodging resistance of large panicle varieties. Rice, belonging to the Poaceae family, is an important cereal crop worldwide and an ideal model system due to its morphological and genetic characteristics (Kim et al., 2014). The PHS of rice is similar to that of other major crops in the Poaceae family and the Fabaceae family, such as maize, wheat, and soybean. Investigations on *G and Seed Dormancy 6* (*SD6*) are examples that extend knowledge on seed dormancy from rice to wheat and soybean (Wang et al., 2018; Xu et al., 2022). Therefore, rice is a good model for seed dormancy research for the Poaceae family, even for the Fabaceae family.

Seed dormancy refers to the phenomenon where viable seeds cannot germinate under suitable environmental conditions such as temperature, water, light, and oxygen. Notably, PHS is closely related to seed dormancy, and varieties with strong dormancy have stronger resistance to PHS. Therefore, enhancing seed dormancy is an effective means of inhibiting PHS. However, to accelerate the breeding process, breeders have eliminated some dormant germplasm during breeding, resulting in high‐yield varieties often experiencing PHS. Thus, cloning seed dormancy genes is essential for ensuring high yields and achieving food security worldwide.

So far, 7 QTLs for seed dormancy have been cloned in rice, including *Sdr4*(*LOC_Os07g39700*) (Sugimoto et al., 2010), *qSD7‐1* (*LOC_Os07g11020*) (Gu et al., 2011), *qSD1‐2* (*LOC_Os01g66110*) (Ye et al., 2015), *qsd‐1‐1* (*LOC_Os01g20030*) (Wang, Lin, et al., 2020), *SD6* (*LOC_Os06g06900*) (Xu et al., 2022), *SDR3.1* (*LOC_Os03g11550*) (Guo et al., 2024), and *qPSR8* (*LOC_os08g42440*) (Fan et al., 2025). However, there is still a lack of understanding of the relationship between different dormant genes. Moreover, research on using cloned dormant genes for improving PHS resistance is still relatively scarce.

Abscisic acid (ABA) and gibberellins (GA) are the primary hormones that antagonize and regulate seed dormancy (Penfield, 2017; Sajeev et al., 2024; Sano & Marion‐Poll, 2021; Shu et al., 2016). ABA promotes seed dormancy, while GA releases seed dormancy. Notably, ABA regulation of dormancy involves its content and signal transduction. Additionally, several factors regulating ABA signal transduction in seeds have recently been identified, such as *PHS8* (Du et al., 2018), *PHS9* (Xu et al., 2019), *SDR3.1* (Guo et al., 2024), *OsFTIP1* (Shen et al., 2024), and *qPSR8* (Fan et al., 2025). The ABA signaling pathway involves many transcription factors, among which *ABI3* and *ABI5* are more critical. *ABI5* enhances seed dormancy (Du et al., 2018; Guo et al., 2024; Zou et al., 2008), and several factors can inhibit the activity of ABI5 or degrade the ABI5 protein to promote seed germination (Du et al., 2018; Guan et al., 2014; Ju et al., 2019; Pan et al., 2020; Piskurewicz et al., 2008; Zhao et al., 2019). In comparison, several factors can promote ABI5 expression and maintain its protein stability to enhance seed dormancy (Hu & Yu, 2014; Yang et al., 2021). Relatively speaking, there is limited research on the regulation of seed dormancy by ABI3. ABI3 interacts with TRAB1 to regulate ABA‐induced transcriptional responses, thus playing an important role in regulating rice seed dormancy (Hobo et al., 1999). The interaction among ABI3, Rc, and OsC1 enhances seed sensitivity to ABA, leading to increased seed dormancy and inhibited PHS (Wang, Deng, et al., 2020). Furthermore, ABI3 can positively regulate seed dormancy by activating the expression of *Sdr4* (Chen et al., 2021), which can positively regulate seed dormancy (Zhao et al., 2022).

In our prior research, the dormancy gene *SDR3.1* was cloned from Mengjialaxiaoli, which negatively regulates seed dormancy and mediates seed dormancy differentiation between *Xian* and *Geng*. SDR3.1 interacts directly with ABI5 and inhibits the activation of *EM1/LEA3* by ABI5/ABI3 (Guo et al., 2024). Therefore, we intend to explore further the relationship between *SDR3.1*, *ABI3*, and *Sdr4* using experimental techniques such as AlphaFold3 prediction, Y2H, BiFC, pull‐down, luciferase (LUC), and transgenic technology. Using gene editing technology to knock out the homologous gene *SDR3.1* in foxtail millet and maize, we can elucidate its common role in seed dormancy of cereal crops and preliminarily explore its molecular mechanism. Moreover, using germplasm resources to analyze the effects of different allele combinations of *SDR3.1* and *Sdr4* on rice dormancy will provide theoretical support and effective gene resources for improving crop PHS traits.

## RESULTS

### SDR3.1 physically interacts with ABI3

Our previous study demonstrated that SDR3.1 represses ABI3 transcriptional activation in rice (Guo et al., 2024). To determine whether SDR3.1 could directly interact with ABI3, we performed AlphaFold3 prediction. The result showed that SDR3.1 could bind tightly to ABI3 (Figure S1a), and their interaction involves a 3‐bond method, with a total of 16 binding sites (Figure S1b,c). This interaction was validated by yeast two‐hybrid (Y2H) assays (Figure 1A), in vitro pull‐down (Figure 1B), and co‐immunoprecipitation (Co‐IP) assays in rice protoplasts (Figure 1D). Furthermore, bimolecular fluorescence complementation (BiFC) assays confirmed that the interaction between SDR3.1 and ABI3 occurred in the nucleus (Figure 1C).

**Figure 1:**
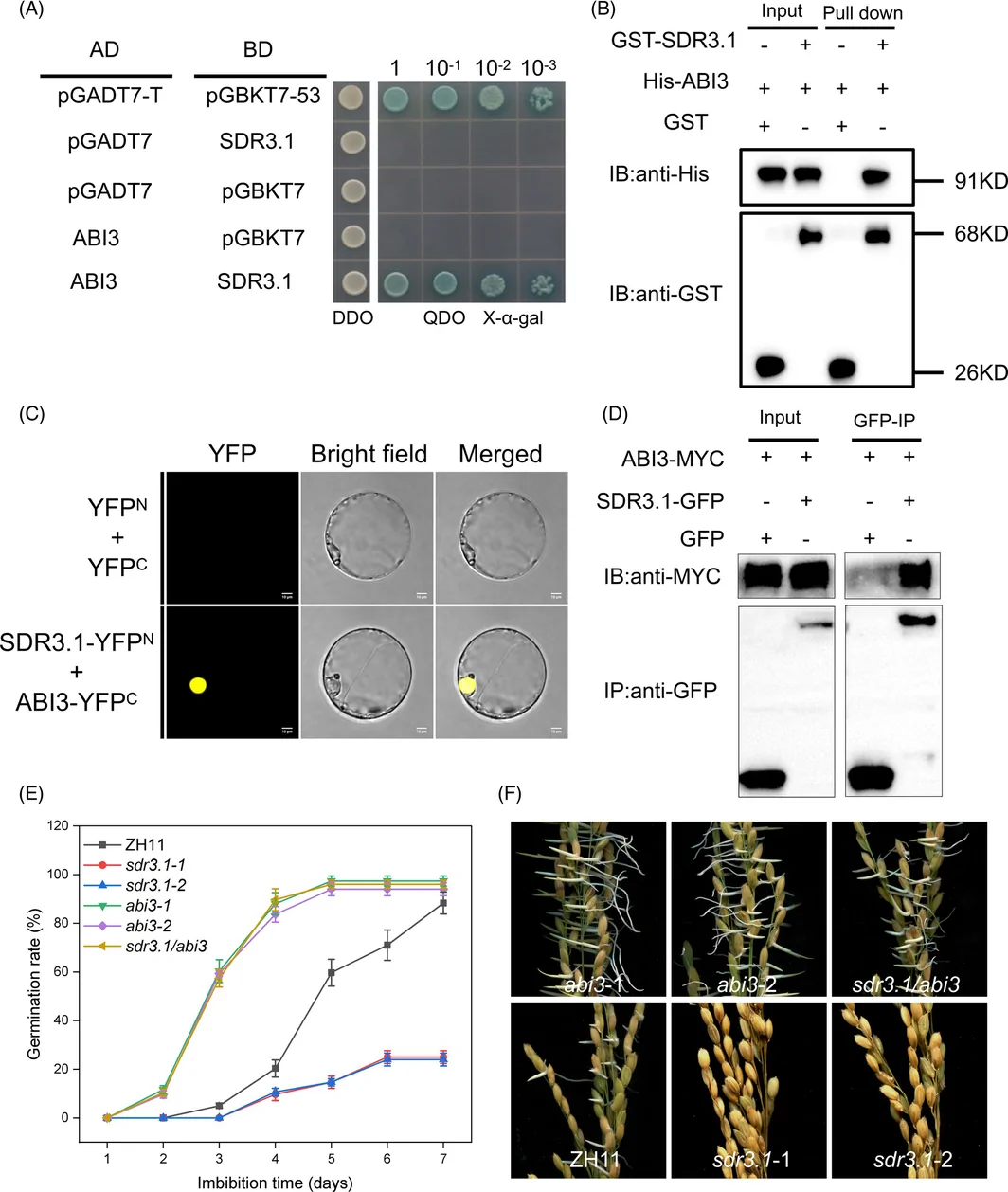
SDR3.1 interacts with ABI3. (A) The Y2H showed that SDR3.1 interacted with ABI3. (B) Pull‐down assays showed that SDR3.1 interacted with ABI3. (C) BiFC assays showed that SDR3.1 interacted with ABI3. Bar = 10 μm. (D) Co‐IP assays showed that SDR3.1 interacted with ABI3. (E) Germination rate of ZH11, *sdr3.1*, *abi3*, and *sdr3.1/abi3* (*n* = 3 independent experiments). (F) PHS phenotypes of ZH11, *sdr3.1*, *abi3*, and *sdr3.1/abi3* 35 DAH. Data are presented as the mean ± SD.

To elucidate the regulatory relationship between *SDR3.1* and *ABI3*, we generated the *sdr3.1/abi3* double mutant in the ZH11 background (Figure S2a,b). Phenotypic analysis revealed that the germination rate of the *sdr3.1/abi3* double mutant was comparable to that of the single mutants *abi3‐1* and *abi3‐2* (Figure 1E,F). The results also implied that SDR3.1 may act upstream of ABI3.

### Transcriptome analysis of *SDR3.1* knockout plants

RNA sequencing (RNA‐seq) was performed to characterize the transcriptomic differences in fresh seeds between ZH11 and the *sdr3.1* mutant. A total of 9886 differentially expressed genes (DEGs) were identified with a significance threshold of *P < 0.05*. Compared with ZH11, 34.0% of these DEGs (3364 genes) were up‐regulated, while 66.0% (6522 genes) were down‐regulated in the *sdr3.1* mutant (Figure 2A).

**Figure 2:**
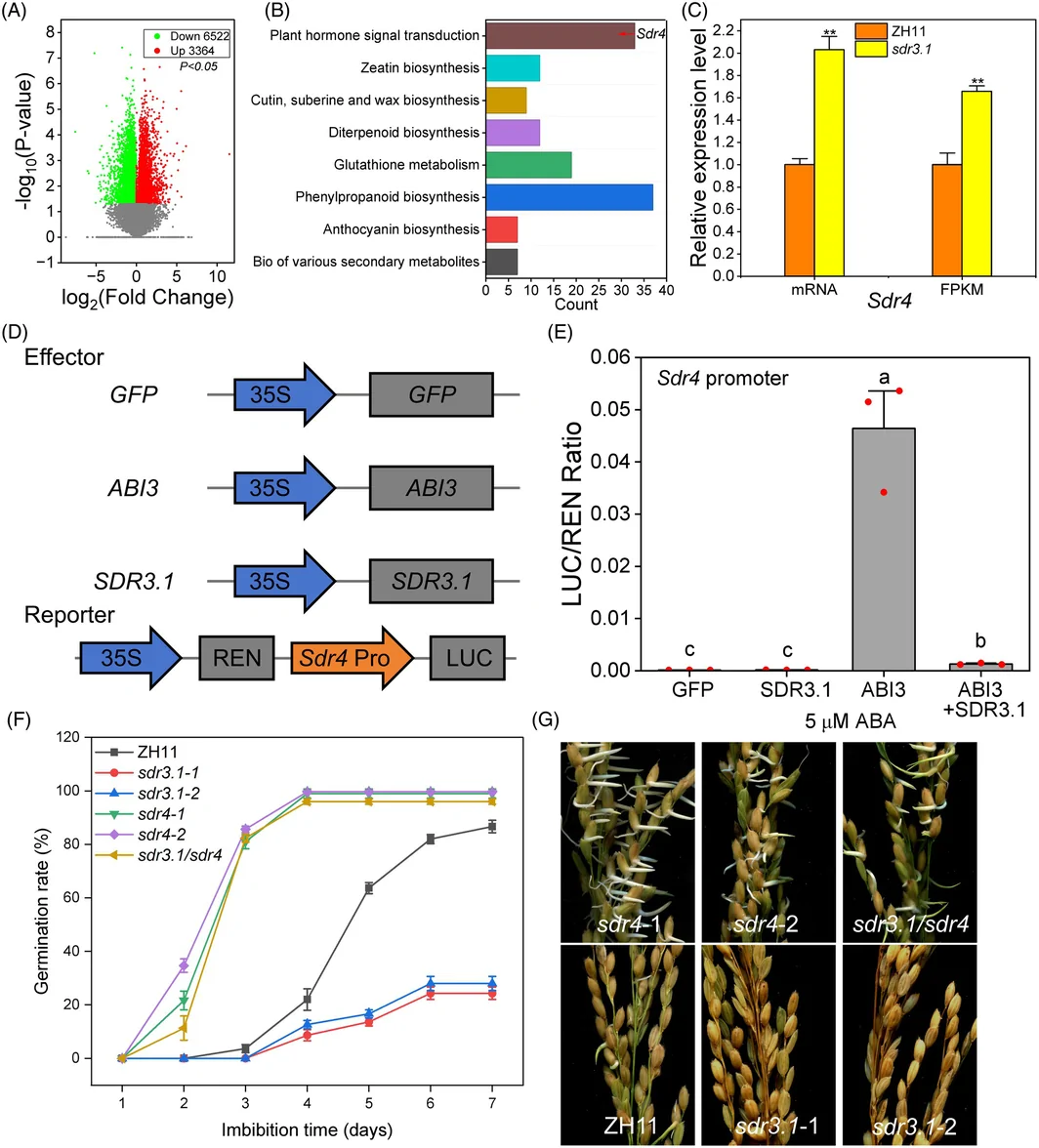
*SDR3.1* negatively regulates the expression of *Sdr4*. (A) Volcano plot of DEGs between *sdr3.1* and ZH11. (B) KEGG pathway enrichment analysis of DEGs. *Sdr4* enrichment in the “plant hormone signal transduction” pathway. (C) The expression of *Sdr4* in *sdr3.1* and ZH11. (D) Pattern diagram of carrier in LUC experiment. (E) SDR3.1 repressed ABI3 to activate *Sdr4* treated with 5 μM ABA. (F) Germination rate of ZH11, *sdr3.1*, *sdr4*, and *sdr3.1/sdr4* (*n* = 3 independent experiments). (G) PHS phenotypes of ZH11, *sdr3.1*, *sdr4*, and *sdr3.1/sdr4* 35 DAH. Data are presented as the mean ± SD.

Kyoto Encyclopedia of Genes and Genomes (KEGG) pathway enrichment analysis revealed that DEGs were significantly enriched in 8 pathways, including plant hormone signal transduction, zeatin biosynthesis, and phenylpropanoid biosynthesis (Figure 2B). Interestingly, the first cloned rice seed dormancy gene *Sdr4* was in plant hormone signal transduction pathway (Figure 2B). Additionally, combining RNA‐seq and qRT‐PCR analyzed, we found that the expression level of *Sdr4* was significantly higher in *sdr3.1* than in ZH11 (Figure 2C). Thus, the results indicated that SDR3.1 may inhibit the expression of *Sdr4*.

### SDR3.1 inhibits the expression of *Sdr4*

To further explore the relationship between *SDR3.1* and *Sdr4*, we performed AlphaFold3 prediction. The result showed that ABI3 could bind to 3 bases in the *Sdr4* promoter (Figure S3a). However, SDR3.1 disrupts the binding capability of ABI3, reducing the binding site to 1 (Figure S3b). Next, the *LUC* gene was ligated to the promoter region of *Sdr4* to construct the reporter plasmid. For trans‐acting experiments, *SDR3.1*, *ABI3*, and green fluorescent protein (*GFP*) genes were expressed under the regulation of the CaMV 35S promoter (Figure 2D). When the samples were treated with 5 μM ABA, the *Sdr4* reporter showed a marked activation effect in the presence of the *ABI3* effector, in contrast to the *GFP* effector used as a control (Figure 2E). Nevertheless, the concurrent expression of *SDR3.1* and *ABI3* impaired the activation of *LUC* expression induced by *ABI3* (Figure 2E). As such, these observations suggest that *SDR3.1* exerts an inhibitory effect on *Sdr4* expression by suppressing the transcriptional activity of *ABI3*.

To further elucidate the regulatory relationship between *SDR3.1* and *Sdr4*, we generated the *sdr3.1/sdr4* double mutant in the ZH11 background (Figure S4a,b). Phenotypic analysis revealed that the germination rate of the *sdr3.1/sdr4* double mutant was comparable to that of the single mutants *sdr4‐1* and *sdr4‐2* (Figure 2F,G). Notably, the results verified that SDR3.1 inhibited the expression of *Sdr4*.

Because SDR3.1 interacted with ABI5 (Guo et al., 2024), ABI5 may also bind to the *Sdr4* promoter like ABI3. The yeast one‐hybrid (Y1H) assay showed that ABI5 could bind to the promoter of *Sdr4* (Figure S5a). The qRT‐PCR results also showed that the expression level of *Sdr4* was significantly reduced in *abi5* compared with ZH11 (Figure S5b). This indicates that ABI5 promotes the expression of *Sdr4*. In addition, we used a LUC experiment to explore the relationship among *SDR3.1*, *ABI5*, and *Sdr4*. To do so, the *LUC* gene was ligated to the promoter region of *Sdr4* to construct the reporter plasmid. *SDR3.1* and *ABI5* were expressed under the regulation of the CaMV 35S promoter (Figure S5c). The concurrent expression of SDR3.1 and ABI5 impaired the activation of LUC expression induced by ABI5 (Figure S5d), indicating that SDR3.1 inhibited the expression of *Sdr4* via repressing the transcriptional activity of ABI5.

Because SDR3.1 interacted with ABI5 and ABI3, we tested whether the 3 genes could form a complex by a yeast three‐hybrid (Y3H) assay. The results showed that ABI5 could interact with ABI3 and the 3 genes could form a complex (Figure 3A). This interaction was further validated by Co‐IP assays in rice protoplasts (Figure 3B). Furthermore, we have created a triple mutation (TM) of *SDR3.1*, *ABI3*, and *ABI5* via CRISPR/Cas9 (Figure S6). The germination rate of the TM was similar to *abi3‐1* (Figure 3C).

**Figure 3:**
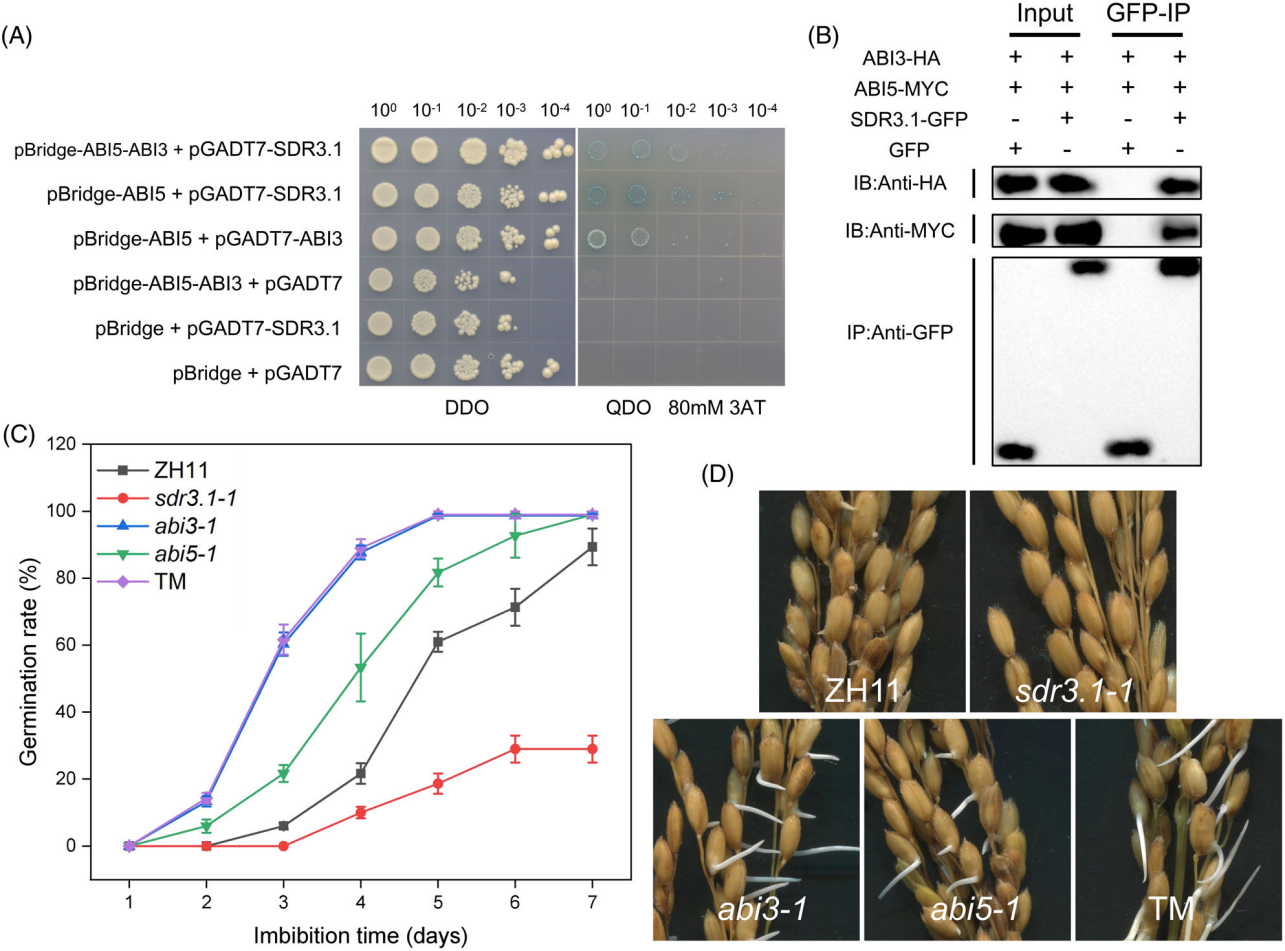
SDR3.1 can form complexes with ABI3 and ABI5. (A) The Y3H showed that SDR3.1 interacted with ABI3 and ABI5. (B) Co‐IP assays showed that SDR3.1 interacted with ABI3 and ABI5. (C) Germination rate of ZH11, *sdr3.1–1*, *abi3‐1*, *abi5‐1*, and TM (*n* = 3 independent experiments). (D) PHS phenotypes of ZH11, *sdr3.1–1*, *abi3‐1*, *abi5‐1*, and TM 35 DAH. Data are presented as the mean ± SD.

In all, these results indicated that SDR3.1 inhibits the expression of *Sdr4*.

### The effect of different allele combinations of *SDR3.1* and *Sdr4* on dormancy

Given that *SDR3.1* and *Sdr4* are major dormant QTLs, we analyzed different allele combinations of *SDR3.1* and *Sdr4* in 273 natural rice populations. Based on the difference bases at positions 305 and 3399, we classified *SDR3.1* into three allele types: m, h, and l (Figure 4A); similarly, based on the difference bases at positions 596 and 606, we classified *Sdr4* into two allele types: n and k (Figure 4B). The average germination rate of h‐n (64.4%) was the highest, followed by those of m‐n (38.9%) and m‐k (14.5%), and the average germination rate of l‐n (7.7%) and l‐k (2.4%) was the lowest (Figure 4C). Correspondingly, we investigated the paddy field PHS situation of different haplotype combinations. The results showed that varieties containing l‐type had the lowest PHS phenomenon, with almost no sprouting (Figure S7). Among the 273 rice accessions, 77 were *Xian*/*indica*, and 77 were *Geng/japonica*. *Xian/indica* was mainly m‐n and m‐k, accounting for 57% and 36%, respectively. *Geng/japonica* was mainly h‐n, accounting for 87% (Figure 4D). Based on the above findings, the best method to improve the PHS resistance of *Geng/japonica* is to replace the h‐type of *SDR3.1* with the l‐type. There are two ways to improve the PHS resistance of *Xian*/*indica*: one is to replace the n‐type of *Sdr4* with the k‐type, and the other is to replace the m‐type of *SDR3.1* with the l‐type.

**Figure 4:**
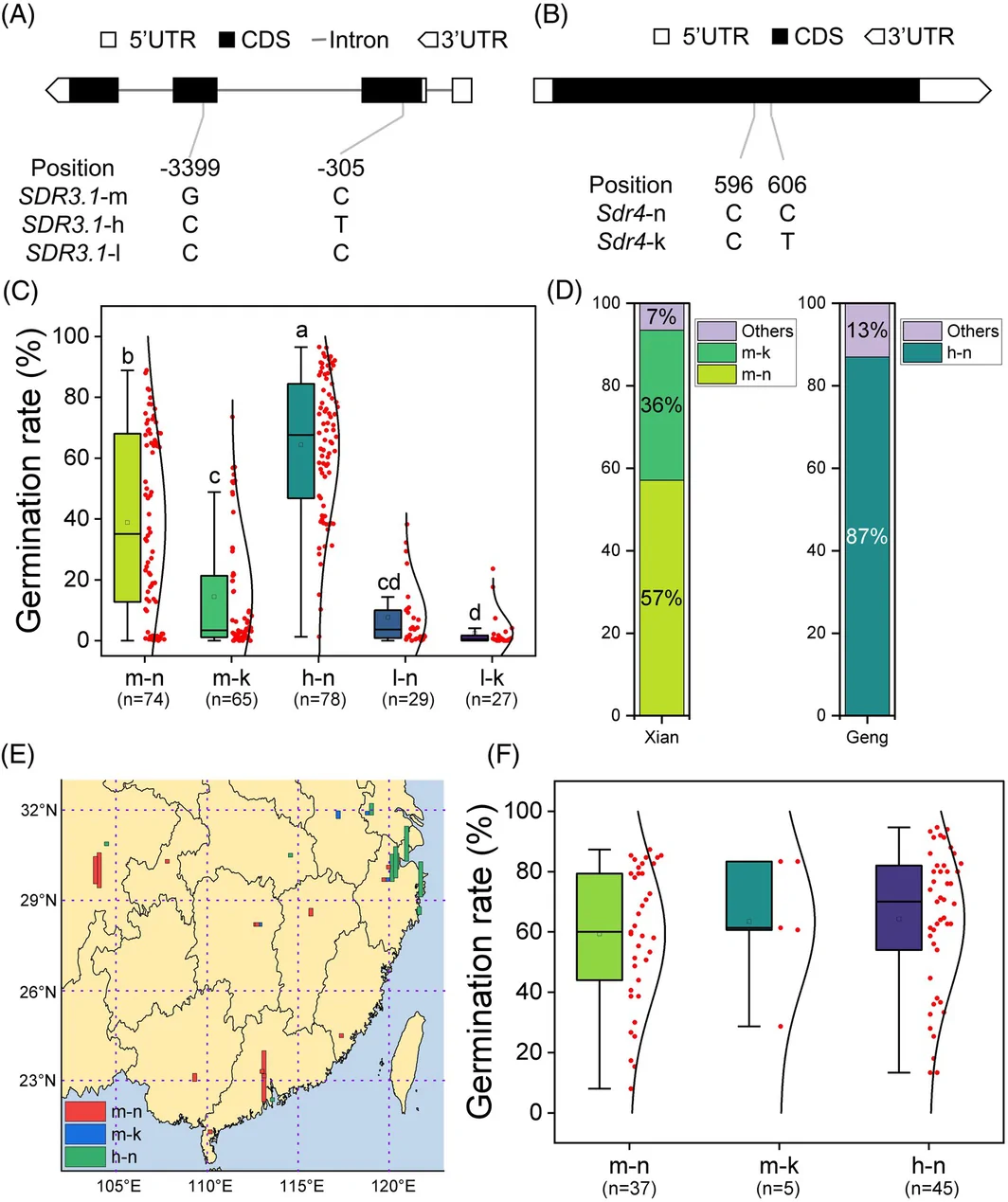
The effect of different allele combinations of *SDR3.1* and *Sdr4* on dormancy. (A) Haplotypes of *SDR3.1* in 273 accessions according to two SNPs in the coding sequence region. (B) Haplotypes of *Sdr4* in 273 accessions according to two SNPs in the coding sequence region. (C) The 7‐day germination rate of the five haplotype combinations (273 rice accessions) 35 DAH. (D) Rice subspecies distribution of each haplotype combination. (E) Geographic distribution of the three haplotype combinations among 87 accessions of core breeding parents. N: North latitude; E: East longitude. (F) The 7‐day germination rate of the three haplotype combinations (87 accessions of core breeding parents) 35 DAH. The lower case letters above the bars indicated significant differences based on ANOVA analysis (*p* < 0.05).

Furthermore, we collected core breeding parents (87 accessions) from southern China's rice‐growing regions (Figure 4E) and conducted germination experiments. These 87 rice varieties are divided into three types, with m‐n accounting for 42.5%, m‐k accounting for 5.8%, and h‐n accounting for 51.7% (Figure 4F). The average germination rates of the three types were 59.3%, 63.5%, and 64.3% (Figure 4F), respectively, all of which showed high germination rates. Among these parents, no l‐type parent showed a low germination rate. Therefore, introducing l‐type allele genes into breeding parents is a feasible method to improve PHS resistance.

### SDR3.1 functions conservatively in major crops of the Poaceae family

Previous research indicated that the *SDR3.1* gene encodes a Mediator of OsbZIP46 deactivation and degradation, which contains an EAR domain, a NINJA_B domain, and a TDBD domain (Guo et al., 2024). Phylogenetic analysis indicated that *SDR3.1* shares an orthologous relationship with *SiSDR3.1* (*Seita.9G490900*) from *Setaria italica* (Figure S8a), recorded as a Ninja‐family protein 1 isoform X1 in the foxtail millet gene repository (https://111.203.21.71:8000/search.html). Moreover, based on the phylogenetic tree (Figure S8a) and BLAST sequence identity results (Figure S8b), two SDR3.1 orthologs were identified within the Poaceae family, namely *ZmSDR3.1* (*Zm00001eb400170*) in Zea mays and *SorSDR3.1* (*SORBI_3001G456400*) in *Sorghum bicolor*.

To verify whether the function of *SDR3.1* in regulating seed dormancy is conserved among Poaceae species, we used the CRISPR/Cas9 system to knock out *SiSDR3.1* in the Ci846 genotype. Sequencing verification confirmed the acquisition of two homozygous mutant lines, *Sisdr3.1‐1* and *Sisdr3.1‐2*, both of which carried a 1‐bp frameshift insertion in the first exon (Figure 5A). Consistent with our expectations, the germination rates of these two mutant lines were significantly lower than that of Ci846 (Figure 5B,C). Additionally, we generated two homozygous mutants in the B104 genotype via CRISPR/Cas9‐mediated genome editing (Figure 5D). Importantly, the *Zmsdr3.1* mutants in maize also displayed a marked decrease in germination rates (Figure 5E,F). Taken together, the results of these transgenic experiments demonstrate that *SDR3.1* plays a conserved role in regulating seed dormancy across Poaceae species.

**Figure 5:**
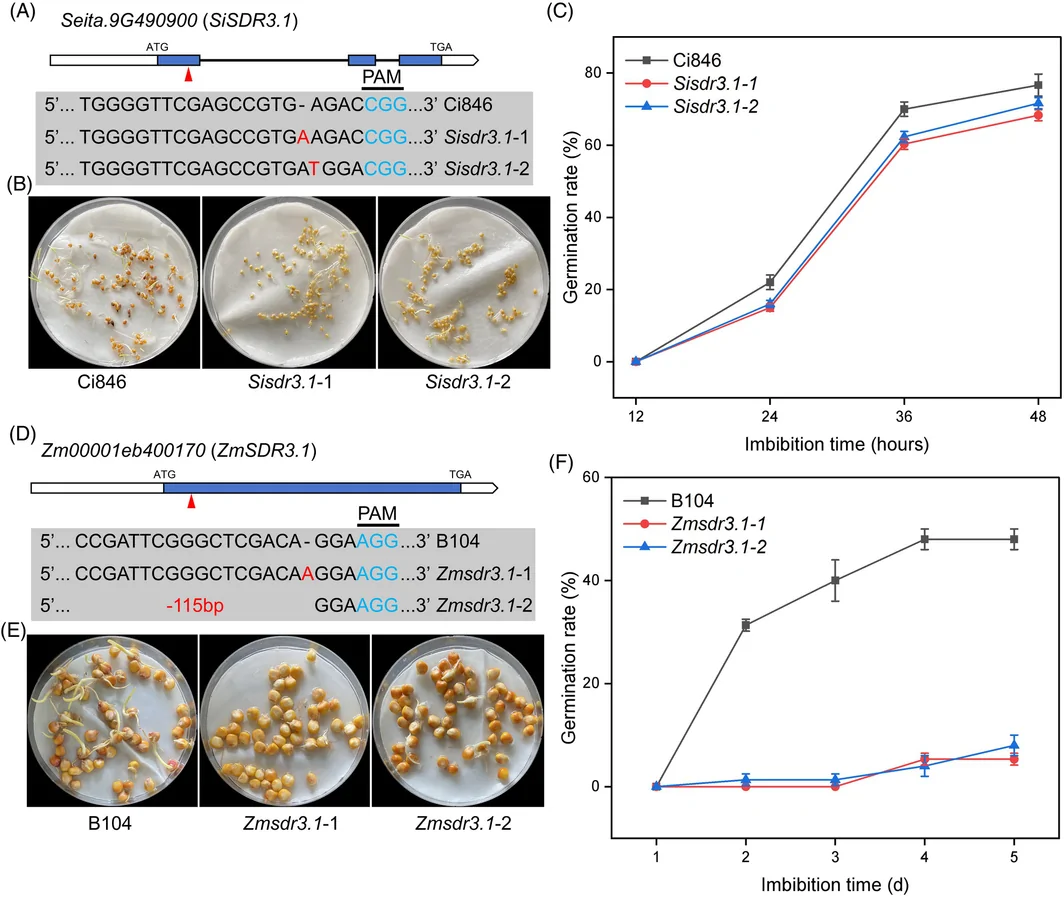
*SDR3.1* regulates seed dormancy in major cereal crops. (A) Generation of *Sisdr3.1‐1* and *Sisdr3.1‐2*. (B) Germination phenotypes of Ci846 and the two mutants. (C) Germination rate of Ci846 and the two mutants (*n* = 3 independent experiments). (D) Generation of *Zmsdr3.1‐1* and *Zmsdr3.1‐2*. (E) Germination phenotypes of B104 and the two mutants. (F) Germination rate of B104 and the two mutants (*n* = 3 independent experiments). Data are presented as the mean ± SD.

To explore whether the molecular mechanism of *SDR3.1* regulating dormancy in different crops is the same, we first found the homologous gene of *ABI3*. Phylogenetic analysis suggests that *ABI3* is orthologous to *SiABI3* (*Seita.5G423900*), *SorABI3* (*Sobic.003G398200*), and *ZmABI3* (*Zm00001eb143690*) (Figure S9a), which all contain a Bfil_C_EcoRII_N_B3 domain (Figure S9b). We also found that SiSDR3.1 could interact with SiABI3 in *Setaria italica* via using Y2H assays (Figure 6A; Figure S10a). Subsequently, the in vivo interaction between the SiSDR3.1 protein and the SiABI3 protein in the nucleus was confirmed by luciferase complementation imaging (LCI) and Co‐Immunoprecipitation(Co‐IP) experiments (Figure 6B,C). In maize, the Y2H results of ZmABI3 (BD) and ZmSDR3.1 (AD) showed that they can interact (Figure S10b), but ZmSDR3.1 (BD) and ZmABI3 (AD) do not interact with each other (Figure 6D). Further, LCI and Co‐IP also prove that ZmSDR3.1 cannot interact with ZmABI3 (Figure 6E,F). Therefore, the above results indicate that *SDR3.1* has different mechanisms in regulating seed dormancy in different crops.

**Figure 6:**
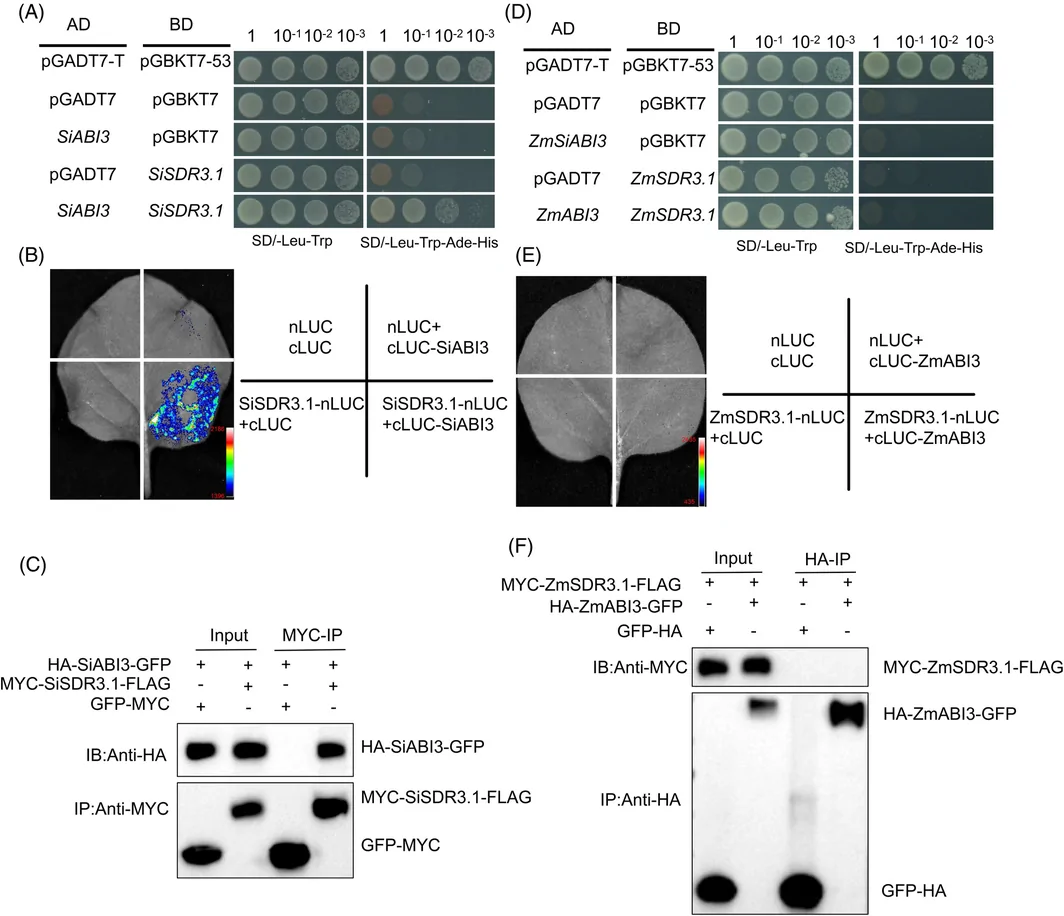
*SDR3.1* has different mechanisms in regulating seed dormancy in different crops. (A) The Y2H showed that SiSDR3.1 interacted with SiABI3. (B) The LCI assay showed that SiSDR3.1 interacted with SiABI3. (C) The Co‐IP assay showed that SiSDR3.1 interacted with SiABI3. (D) The Y2H assay showed that ZmSDR3.1 did not interact with ZmABI3. (E) The LCI assay showed that ZmSDR3.1 did not interact with ZmABI3. (F) The Co‐IP assay showed that ZmSDR3.1 did not interact with ZmABI3.

## DISCUSSION

From seed dormancy to germination is an initial and important stage; this transition is governed by complex regulatory networks (Wang, Zhang, et al., 2025). Specifically, seed dormancy—a quantitative trait associated with plant domestication—is jointly controlled by multiple genes and modulated by diverse environmental factors (Graeber et al., 2012; Penfield & MacGregor, 2017; Xu et al., 2022). This characteristic renders the identification of seed dormancy‐related genes extremely challenging. *Sdr4*, which positively regulates rice seed dormancy, is the first gene cloned from the indica cultivar Kasalath (Sugimoto et al., 2010). ABI3 can directly bind to the *Sdr4* promoter and thereby activate its transcription (Chen et al., 2021). In our prior research, SDR3.1 interacts directly with ABI5 and inhibits the activation of *EM1/LEA3* by ABI5/ABI3 (Guo et al., 2024). In this study, we found the relationship between two seed dormancy QTLs, *SDR3.1* and *Sdr4*, and provide AI, molecular, biochemical, and genetic evidence supporting the signaling network in which they work alongside ABI5 and ABI3. Thus, we propose the following network: SDR3.1, ABI3, and ABI5 form a complex; then SDR3.1 inhibits the transcriptional activation function of ABI3 and ABI5, leading to decreased expression of the downstream gene *Sdr4* of ABI3/ABI5, ultimately resulting in seed germination (Figure 7).

**Figure 7:**
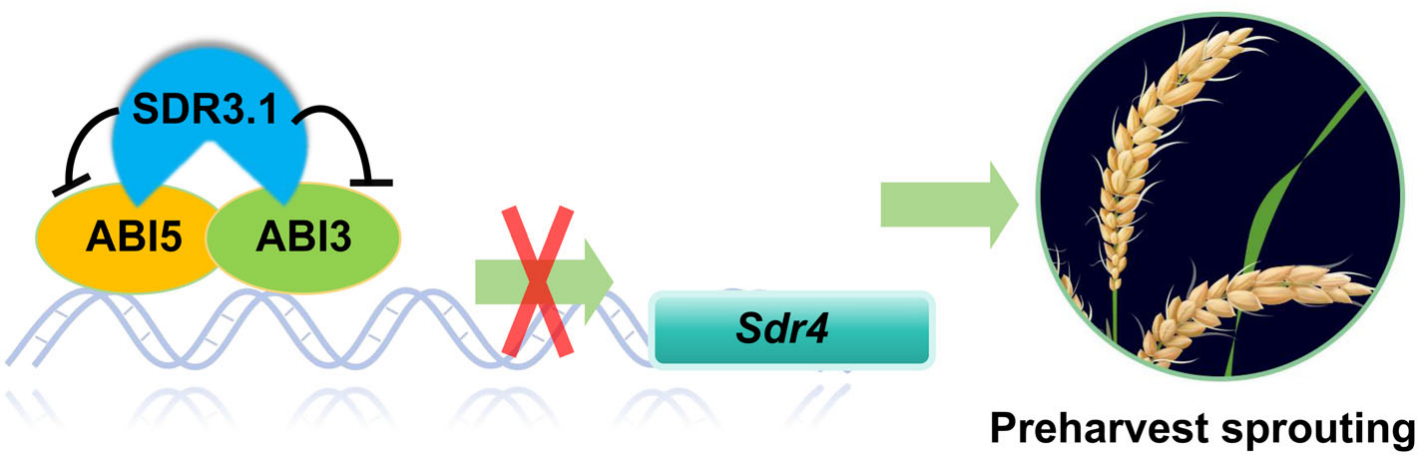
Proposed working model for *SDR3.1* in rice. SDR3.1, ABI3, and ABI5 interact to form a complex. In contrast, SDR3.1 inhibits ABI3‐ and ABI5‐mediated activation of *Sdr4*, ultimately leading to seed germination.

Domestication can be considered the earliest form of breeding (Wang et al., 2018); this process has resulted in the loss of many elite haplotype genes. Therefore, identifying and utilizing elite haplotype genes has become a hot topic in current agricultural research. For example, as a high‐NUE haplotype, OsGATA8‐H promoted nitrogen uptake and increased the proportion of productive tillers (Wu et al., 2024). The elite allele *SBRR1‐R* is preferentially distributed in indica rice varieties; its introduction into japonica rice varieties notably alleviated yield loss under conditions of severe sheath blight disease stress (Feng et al., 2025). Introducing the elite *CSP* haplotype of *Aegilops tauschii* into wheat could significantly increase grain yields (Wang, Guo, et al., 2025). In addition, this could help identify a large number of elite haplotype genes in areas such as crop drought tolerance (Hou et al., 2025; Li, Yang, et al., 2025; Liu et al., 2025; Sinha et al., 2020; Sun et al., 2022; Xiang et al., 2025), grain shape (Ang et al., 2025; He et al., 2025; Zhao et al., 2025), heat stress tolerance (Lu et al., 2024; Zhang et al., 2022), cold tolerance (Gao et al., 2025; Zeng et al., 2025), seed dormancy (Guo et al., 2024; Zhang et al., 2026), etc. However, a strong dormancy haplotype may lead to poor field emergence and thus hinder the popularization of direct seeding cultivation.

Therefore, the combined analysis of multiple dormant gene haplotypes can provide more choices for PHS resistance breeding, just like the trait regulatory haplotypes (TRHs) established in the study of heat‐tolerant genes (Li, Zhu, et al., 2025). In this study, we found the effects of different allele combinations of *SDR3.1* and *Sdr4* on rice dormancy and proposed strategies for improving PHS resistance in indica and japonica rice; furthermore, in the process of half a century of southern and northern breeding, to accelerate the breeding process and ensure rapid germination of seeds, breeders have eliminated dormant germplasm. As a result, elite dormant haplotypes are extremely rare in current core breeding parents. Therefore, introducing elite dormant haplotypes is an effective strategy for improving PHS resistance of core breeding parents. However, as recently reported, maintaining an appropriate level of bioactive GA is key to improving rice stress tolerance (Guo et al., 2025). As such, further research is required to clarify the optimal level of seed dormancy that is beneficial for both PHS resistance and direct‐seeded cultivation (Xu et al., 2025).

A few seed dormancy genes exhibited parallel selection across different families. *G* gene maintains conserved regulatory roles in modulating seed dormancy across soybean, rice, and Arabidopsis (Wang et al., 2018), while *SD6* is conserved in rice and wheat (Xu et al., 2022). In this study, *SDR3.1* negatively regulates seed dormancy in cereal crops such as rice, foxtail millet, and maize. The identification of parallel selection genes will facilitate the domestication of new crops (Wang et al., 2018). Interestingly, SDR3.1 has different molecular mechanisms for regulating seed dormancy in different crops. SDR3.1 in rice and foxtail millet regulates seed dormancy through ABI3, while SDR3.1 in maize does not. We speculate that such divergence in molecular mechanisms might be related to the homology relationship: Compared to maize, foxtail millet has a closer homologous relationship with rice (Figures S8a, S9a). Moreover, previous studies have shown that *Sdr4* in Arabidopsis negatively regulates seed dormancy (Liu et al., 2020), while *Sdr4* in rice positively regulates seed dormancy (Sugimoto et al., 2010). Such divergence in function might be related to different molecular mechanisms and sequence variation in their coding sequences (Liu et al., 2020). Therefore, the different molecular mechanisms of *SDR3.1* might be related to the function of downstream genes; this requires further research.

## METHODS

### Plant material and growth conditions

The mutants *sdr3.1‐*1 and *sdr3.1‐*2 were obtained from previous research (Guo et al., 2024). Foxtail millet transgenic plants (*sisdr3.1‐1* and *sisdr3.1‐2*) were obtained from the Ci846 variety. Maize transgenic plants (*zmsdr3.1‐1* and *zmsdr3.1‐2*) were generated from the B104 variety. All rice transgenic plants were developed using the ZH11 genotype as the background. For haplotype combination analysis, a population consisting of 273 rice accessions (Guo et al., 2024) and 87 core breeding parents (materials collected and preserved by our research group, Table S1) was used. In terms of field trials, foxtail millet was cultivated in the experimental field of Shanxi Agricultural University, China (37°25′N, 112°35′E). Meanwhile, maize and rice were grown in Hangzhou and Hainan, respectively.

### Evaluation of seed germination

Rice seed germination was performed following the method previously reported (Guo et al., 2024). For foxtail millet germination assays, 100 seeds were placed in each 90‐mm petri dish lined with two layers of filter paper, and these dishes were then incubated in a dark chamber at 25°C for 48 h to facilitate seed germination. For maize, 50 seeds were placed in each 150‐mm petri dish covered with two layers of filter paper, and the dishes were incubated in a dark incubator at 25°C for 5 days to induce germination.

### AI predicts interaction results

On the AlphaFold3 website (https://alphafoldserver.com/), we input the gene sequence to be validated for prediction. We then opened the prediction results using Visual Studio Code software and selected model_0 for binding site analysis.

### Y1H assay

The CDS of *ABI5* was cloned into the pB42AD vector, while the promoter region of *Sdr4* was inserted into the pLacz vector. These two recombinant plasmids were co‐transformed into the EGY48 yeast strain, which was then cultured on SD‐Ura‐Trp selective medium at 28°C for 3 days. Subsequently, several colonies were randomly picked and spotted onto SD‐Leu‐Trp‐His‐Ade/X‐gal medium supplemented with 2% (w/v) galactose, 1% (w/v) raffinose, 1 × salt buffer (7 g/L Na_2_HPO_4_·PH_2_O, 3 g/L Na_2_HPO_4_, pH 7.0), and 80 mg/L 5‐bromo‐4‐chloro‐3‐indolyl‐β‐d‐galactopyranoside acid and incubated for 1 day.

### Y2H assay

The CDS of *SDR3.1* or *ABI3* was cloned into the pGBKT7 or pGADT7 vector, respectively. The recombinant vectors were then co‐transformed into the Y2Hgold yeast strain, which was cultured on SD‐Leu‐Trp medium at 28°C for 2–4 days. Subsequently, several colonies were randomly selected and spotted onto SD‐Leu‐Trp‐His‐Ade/X‐gal medium, followed by incubation for 3 days.

### Y3H assay

The CDS of *SDR3.1* was inserted into the pGADT7 vector, while the CDS of *ABI3* and *ABI5* was separately integrated into the pBridge backbone. These recombinant constructs were co‐transformed into the AH109 yeast strain and cultivated on SD‐Leu‐Trp medium at 28°C for 3 days. Following growth, independent single colonies were randomly collected and inoculated onto two types of selective media containing X‐α‐gal, namely SD‐Trp‐Leu‐His‐Ade and SD‐Trp‐Leu‐His‐Ade‐Met. All plates were finally incubated for an additional 3 days.

### Pull‐down assay

The SDR3.1 gene cloned from rice cDNA was linked to the pGEX‐4 T‐1 vector. The ABI3 gene cloned from rice cDNA was linked to the pET28a vector to create a fusion protein with a His tag. A pull‐down assay was performed using Glutathione Agarose beads (Solarbio, P2020). The mouse monoclonal anti‐GST, anti‐His, and HRP‐goat anti‐mouse antibodies were obtained from Proteintech or YEASEN at a dilution of 1:10000 (YEASEN: 30901ES50; Proteintech: 66005‐1‐Ig and SA00001‐1).

### BiFC assay

The CDS of *SDR3.1* was ligated into the pSPYCE vector, and the CDS of *ABI3* was inserted into the pSPYNE vector to construct recombinant plasmids. The two recombinant plasmids were then co‐transfected into rice protoplasts. Finally, a Zeiss LSM 710 NLO confocal microscope was used to detect fluorescence signals to verify their interaction.

### Co‐IP assay

The CDS of *SDR3.1* was ligated to pCAMBIA1305 containing a GFP tag, and the CDS of *ABI3* was ligated to pCAMBIA1300 containing a MYC tag. The combined plasmids were introduced into rice protoplasts and incubated for 22 h. Co‐IP assay was performed using GFP‐Nanoab‐Agarose (Lablead, GNA‐250‐5K). The mouse monoclonal anti‐GFP, rabbit monoclonal anti‐MYC, HRP‐goat anti‐rabbit antibodies, and HRP‐goat anti‐mouse antibodies were obtained from Roche, Abclonal, or Proteintech at a dilution of 1:5000 (Roche: 11814460001; Abclonal: AE070 and AS003; Proteintech: SA00001‐2).

### LCI assay

The CDS of *SiSDR3.1* or *ZmSDR3.1* was ligated to nLUC, and the CDS of *SiABI3* or *ZmSDR3.1* was ligated to cLUC. The two plasmids of *Si* or *Zm* were transformed into the EHA105 *Agrobacterium* strain. Mix nLUC or cLUC fusion plasmids and infiltrate them into tobacco leaves. Three days after inoculation, the luciferase luminescence signal was imaged using a live plant imaging system (Tanon 5200).

### Transient transactivation assay

We constructed the pGreenII 0800‐LUC recombinant vector for the *Sdr4* promoter to form the reporter vector and constructed the pGreenII 62‐SK recombinant vectors for *ABI3*, *ABI5*, *SDR3.1*, and *GFP* to form the effector vector. We then transferred the recombinant vector into rice protoplasts, treated them with 5 μM ABA at 28°C for 22 h, and detected LUC activity.

### Expression analysis

Total RNA was extracted using a MolPure® Plant Plus RNA Kit (YEASEN, 19292ES50). RNA reverse transcription was performed using TaqMan™ MicroRNA (Applied Biosystems: 4366596). qRT‐PCR was performed with ABI QuantStudio6 FLEX Q6.

### RNA‐seq analysis

Seeds of ZH11 and *sdr3.1* at 35 days after heading were harvested and immediately frozen in liquid nitrogen. The cDNA libraries were constructed in accordance with Illumina standard protocols and sequenced on the Illumina NovaSeq 6000 platform by Biorun (Wuhan, China). Thresholds of *P < 0.05* and |log2foldchange| >1 were set to screen for significantly differentially expressed genes. The clusterProfiler R package (version 3.8.1) was used for GO enrichment and KEGG enrichment analyses.

## Author Contributions

HP and SZ designed the research; GN, AR, XM, LY, RZ, JJ, CB, HX, HS, SG, JG, XL, WL, ZF, ZY and TS performed the experiments; SZ and GN analyzed the results and wrote the manuscript.

## Conflict of Interest

The authors declare that they have no conflict of interest.

## Supporting information


**Figure S1.** AI predicts the interaction between SDR3.1 and ABI3. (a) SDR3.1 and ABI3 interaction pattern diagram. (b, c) The binding sites of SDR3.1 and ABI3. The red arrow indicates the specific combination position.
**Figure S2.** The mutant sites of the *sdr3.1/abi3* double knockout line. (a) *SDR3.1* mutant sites of *sdr3.1/abi3* double knockout line; red arrows indicated knockout sites. (b) *ABI3* mutant sites of *sdr3.1/abi3* double knockout line; red arrows indicated knockout sites.
**Figure S3.** AI predictions of the interaction between SDR3.1, ABI3, and *Sdr4*. (a) ABI3 binds to three sites in the *Sdr4* promoter region. (b) SDR3.1 reduces the binding sites of ABI3 to the *Sdr4* promoter region.
**Figure S4.** The mutant sites of the *sdr3.1/sdr4* double knockout line. (a) *SDR3.1* mutant sites of *sdr3.1/sdr4* double knockout line; red arrows indicated the knockout sites. (b) *Sdr4* mutant sites of *sdr3.1/sdr4* double knockout line; red arrows indicated the knockout sites.
**Figure S5.** SDR3.1 inhibits the expression level of *Sdr4* by ABI5. (a) Yeast one‐hybrid assay showed that *ABI5* bound to the *Sdr4* promoter. (b) Expression levels of *Sdr4* in *abi5* and ZH11. (c) Schematic of the effectors and reporter used in the transient transactivation assays. (d) SDR3.1 repressed ABI5 to activate *Sdr4* in response to 5 μM ABA.
**Figure S6.** The mutant sites of the TM line. (a) *SDR3.1* mutant sites of the TM knockout line; red arrows indicated the knockout sites. (b) *ABI3* mutant sites of the TM knockout line; red arrows indicated the knockout sites. (c) *ABI5* mutant sites of the TM knockout line; red arrows indicated the knockout sites.
**Figure S7.** PHS of different haplotype combinations. Five varieties were investigated for each combination, and the survey was conducted 4 days after rain.
**Figure S8.** Homologous analysis of *SDR3.1*. (a) Evolutionary tree analysis of *SDR3.1*. (b) Multi‐sequence alignment result of *SDR3.1*.
**Figure S9.** Homologous analysis of *ABI3*. (a) Evolutionary tree analysis of *ABI3*. (b) Multi‐sequence alignment result of *ABI3*.
**Figure S10.** Y2H verification of SDR3.1 interaction proteins in foxtail millet and maize. (a) The Y2H showed that SiABI3 interacted with SiSDR3.1. (b) The Y2H assay showed that ZmABI3 interacted with ZmSDR3.1.
**Table S1.** Germination rate of core parents.

## Data Availability

The data that support the findings of this study are available on request from the corresponding author. The data are not publicly available due to privacy or ethical restrictions.

## References

[tpj71141-bib-0001] Ang, Y. , Li, B.X. , Shen, J.M. , Li, J.H. , Zhao, Y.Y. , Li, T.H. et al. (2025) The grain size 8‐grain length 10 module controls the grain size in rice by regulating the expression of GA‐ and CTK‐related genes. The Plant Journal, 122(5), e70247. 10.1111/tpj.70247 40455889

[tpj71141-bib-0002] Chen, W.Q. , Wang, W. , Lyu, Y.S. , Wu, Y.W. , Huang, P.L. , Hu, S.K. et al. (2021) OsVP1 activates Sdr4 expression to control rice seed dormancy via the ABA signaling pathway. The Crop Journal, 9(1), 11.

[tpj71141-bib-0003] Du, L. , Xu, F. , Fang, J. , Gao, S.P. , Tang, J.Y. , Fang, S. et al. (2018) Endosperm sugar accumulation caused by mutation of PHS8/ISA1 leads to pre‐harvest sprouting in rice. The Plant Journal, 95(3), 545–556. 10.1111/tpj.13970 29775500

[tpj71141-bib-0004] Fan, X.W. , Gao, F.Y. , Liu, Y.X. , Huang, W. , Yang, Y. , Luo, Z.L. et al. (2025) The transcription factor CCT30 promotes rice preharvest sprouting by regulating sugar signalling to inhibit the ABA‐mediated pathway. Plant Biotechnology Journal, 23(2), 579–591. 10.1111/pbi.14521 PMC11772322 39622700

[tpj71141-bib-0005] Feng, Z.M. , Gao, P. , Wang, G.D. , Kang, H.X. , Zhao, J.H. , Xie, W.Y. et al. (2025) Natural variation in SBRR1 shows high potential for sheath blight resistance breeding in rice. Nature Genetics, 57(8), 2004–2016. 10.1038/s41588-025-02281-4 PMC12339379 40738998

[tpj71141-bib-0006] Gao, S.L. , Li, J. , Zeng, Y.W. , Li, H.H. , Guo, Z.H. , Guo, H.F. et al. (2025) CTB6 confers cold tolerance at the booting stage by maintaining tapetum development in Rice. Advanced Science, 12(10), e2411357. 10.1002/advs.202411357 PMC11905004 39840555

[tpj71141-bib-0007] Graeber, K. , Nakabayashi, K. , Miatton, E. , Leubner‐Metzger, G. & Soppe, W.J. (2012) Molecular mechanisms of seed dormancy. Plant, Cell & Environment, 35(10), 1769–1786. 10.1111/j.1365-3040.2012.02542.x 22620982

[tpj71141-bib-0008] Gu, X.Y. , Foley, M.E. , Horvath, D.P. , Anderson, J.V. , Feng, J. , Zhang, L. et al. (2011) Association between seed dormancy and pericarp color is controlled by a pleiotropic gene that regulates abscisic acid and flavonoid synthesis in weedy red rice. Genetics, 189(4), 1515–1524. 10.1534/genetics.111.131169 PMC3241415 21954164

[tpj71141-bib-0009] Guan, C.M. , Wang, X.C. , Feng, J. , Hong, S.L. , Liang, Y. , Ren, B. et al. (2014) Cytokinin antagonizes abscisic acid‐mediated inhibition of cotyledon greening by promoting the degradation of abscisic acid insensitive5 protein in Arabidopsis. Plant Physiology, 164(3), 1515–1526. 10.1104/pp.113.234740 PMC3938637 24443524

[tpj71141-bib-0010] Guo, N.H. , Tang, S.J. , Wang, Y.K. , Chen, W. , An, R.H. , Ren, Z.L. et al. (2024) A mediator of OsbZIP46 deactivation and degradation negatively regulates seed dormancy in rice. Nature Communications, 15(1), 1134. 10.1038/s41467-024-45402-z PMC10850359 38326370

[tpj71141-bib-0011] Guo, S.Q. , Chen, Y.X. , Ju, Y.L. , Pan, C.Y. , Shan, J.X. , Ye, W.W. et al. (2025) Fine‐tuning gibberellin improves rice alkali‐thermal tolerance and yield. Nature, 639(8053), 162–171. 10.1038/s41586-024-08486-7 39880957

[tpj71141-bib-0012] He, F. , Du, D.J. , Chen, Z.Y. , Ma, L. , Liu, Y.J. , Li, Z.J. et al. (2025) A natural variation in the promoter of TaGDSL‐7D contributes to grain weight and yield in wheat. Plant Biotechnology Journal, 23(9), 3851–3863. 10.1111/pbi.70204 PMC12392936 40522813

[tpj71141-bib-0013] Hobo, T. , Kowyama, Y. & Hattori, T. (1999) A bZIP factor, TRAB1, interacts with VP1 and mediates abscisic acid‐induced transcription. Proceedings of the National Academy of Sciences of the United States of America, 96(26), 15348–15353. 10.1073/pnas.96.26.15348 PMC24822 10611387

[tpj71141-bib-0014] Hodson, R. (2017) Food security. Nature, 544(7651), S5. 10.1038/544S5a 28445447

[tpj71141-bib-0015] Hou, X.Y. , Guo, C.J. , Qiao, Y.J. , Wang, J.L. , Wang, Z.Y. , Zhang, J.Q. et al. (2025) Wheat miRNA TaMIR5062‐5A targets calmodulin TaCML31 that cooperates with MYB member TaMYB77 to modulate drought and salt responses. Plant, Cell & Environment, 48(10), 7562–7587. 10.1111/pce.70038 40613268

[tpj71141-bib-0016] Hu, Y.R. & Yu, D.Q. (2014) BRASSINOSTEROID INSENSITIVE2 interacts with ABSCISIC ACID INSENSITIVE5 to mediate the antagonism of brassinosteroids to abscisic acid during seed germination in Arabidopsis. Plant Cell, 26(11), 4394–4408. 10.1105/tpc.114.130849 PMC4277219 25415975

[tpj71141-bib-0017] Ju, L. , Jing, Y.X. , Shi, P.T. , Liu, J. , Chen, J.S. , Yan, J.J. et al. (2019) JAZ proteins modulate seed germination through interaction with ABI5 in bread wheat and Arabidopsis. New Phytologist, 223(1), 246–260. 10.1111/nph.15757 30802963

[tpj71141-bib-0018] Kim, S.T. , Kim, S.G. , Agrawal, G.K. , Kikuchi, S. & Rakwal, R. (2014) Rice proteomics: a model system for crop improvement and food security. Proteomics, 14(4–5), 593–610. 10.1002/pmic.201300388 24323464

[tpj71141-bib-0019] Li, P.C. , Zhu, T.Z. , Wang, Y.Y. , Zhang, X.M. , Yang, X.Y. , Fang, S. et al. (2025) Natural variation in a cortex/epidermis‐specific transcription factor bZIP89 determines lateral root development and drought resilience in maize. Science Advances, 11(15), eadt1113. 10.1126/sciadv.adt1113 PMC11988453 40215297

[tpj71141-bib-0020] Li, W. , Yang, K. , Hu, C.F. , Abbas, W. , Zhang, J. , Xu, P.K. et al. (2025) A natural gene on‐off system confers field thermotolerance for grain quality and yield in rice. Cell, 188(14), 3661–3678. 10.1016/j.cell.2025.04.011 40311617

[tpj71141-bib-0021] Liu, F. , Zhang, H. , Ding, L. , Soppe, W.J.J. & Xiang, Y. (2020) REVERSAL OF RDO5 1, a homolog of Rice seed Dormancy4, interacts with bHLH57 and controls ABA biosynthesis and seed dormancy in Arabidopsis. Plant Cell, 32(6), 1933–1948. 10.1105/tpc.20.00026 PMC7268807 32213638

[tpj71141-bib-0022] Liu, X.B. , Li, J.P. , Zhang, C.J. , Zhao, D.Y. , Peng, X. , Yang, Q. et al. (2025) An elite allele TaDT1‐a(hapI) enhances drought tolerance via mediating autophagic pathways in wheat. Nature Communications, 16(1), 6563. 10.1038/s41467-025-61943-3 PMC12267691 40670373

[tpj71141-bib-0023] Lu, F.F. , Jiao, G.A. , Qiu, J.H. , Zhao, S.L. , Zhao, F.L. , Wang, P. et al. (2024) A molecular module improves rice grain quality and yield at high temperatures. National Science Review, 12(2), nwae416. 10.1093/nsr/nwae416 PMC11759936 39868077

[tpj71141-bib-0024] Pan, J.J. , Hu, Y.R. , Wang, H.P. , Guo, Q. , Chen, Y.N. , Howe, G.A. et al. (2020) Molecular mechanism underlying the synergetic effect of Jasmonate on abscisic acid signaling during seed germination in Arabidopsis. Plant Cell, 32(12), 3846–3865. 10.1105/tpc.19.00838 PMC7721325 33023956

[tpj71141-bib-0025] Penfield, S. (2017) Seed dormancy and germination. Current Biology, 27(17), R874–R878. 10.1016/j.cub.2017.05.050 28898656

[tpj71141-bib-0026] Penfield, S. & MacGregor, D.R. (2017) Effects of environmental variation during seed production on seed dormancy and germination. Journal of Experimental Botany, 68(4), 819–825. 10.1093/jxb/erw436 27940467

[tpj71141-bib-0027] Piskurewicz, U. , Jikumaru, Y. , Kinoshita, N. , Nambara, E. , Kamiya, Y. & Lopez‐Molina, L. (2008) The gibberellic acid signaling repressor RGL2 inhibits Arabidopsis seed germination by stimulating abscisic acid synthesis and ABI5 activity. Plant Cell, 20(10), 2729–2745. 10.1105/tpc.108.061515 PMC2590721 18941053

[tpj71141-bib-0028] Sajeev, N. , Koornneef, M. & Bentsink, L. (2024) A commitment for life: decades of unraveling the molecular mechanisms behind seed dormancy and germination. Plant Cell, 36(5), 1358–1376. 10.1093/plcell/koad328 PMC11062444 38215009

[tpj71141-bib-0029] Sano, N. & Marion‐Poll, A. (2021) ABA metabolism and homeostasis in seed dormancy and germination. International Journal of Molecular Sciences, 22(10), 5069. 10.3390/ijms22105069 PMC8151144 34064729

[tpj71141-bib-0030] Shen, J. , Zhang, L. , Wang, H.Y. , Guo, J.Z. , Li, Y.C. , Tan, Y.Y. et al. (2024) The phosphatidylethanolamine‐binding proteins OsMFT1 and OsMFT2 regulate seed dormancy in rice. Plant Cell, 36(9), 3857–3874. 10.1093/plcell/koae211 PMC11371141 39041489

[tpj71141-bib-0031] Shu, K. , Liu, X.D. , Xie, Q. & He, Z.H. (2016) Two faces of one seed: hormonal regulation of dormancy and germination. Molecular Plant, 9(1), 34–45. 10.1016/j.molp.2015.08.010 26343970

[tpj71141-bib-0032] Sinha, P. , Singh, V.K. , Saxena, R.K. , Khan, A.W. , Abbai, R. , Chitikineni, A. et al. (2020) Superior haplotypes for haplotype‐based breeding for drought tolerance in pigeonpea (Cajanus cajan L.). Plant Biotechnology Journal, 18(12), 2482–2490. 10.1111/pbi.13422 PMC7680530 32455481

[tpj71141-bib-0033] Sugimoto, K. , Takeuchi, Y. , Ebana, K. , Miyao, A. , Hirochika, H. , Hara, N. et al. (2010) Molecular cloning of Sdr4, a regulator involved in seed dormancy and domestication of rice. Proceedings of the National Academy of Sciences of the United States of America, 107(13), 5792–9797. 10.1073/pnas.0911965107 PMC2851884 20220098

[tpj71141-bib-0034] Sun, X.M. , Xiong, H.Y. , Jiang, C.H. , Zhang, D.M. , Yang, Z.L. , Huang, Y.P. et al. (2022) Natural variation of DROT1 confers drought adaptation in upland rice. Nature Communications, 13(1), 4265. 10.1038/s41467-022-31844-w PMC9308802 35871266

[tpj71141-bib-0035] Wang, J. , Deng, Q.W. , Li, Y.H. , Yu, Y. , Liu, X. , Han, Y.F. et al. (2020) Transcription factors Rc and OsVP1 coordinately regulate preharvest sprouting tolerance in red pericarp Rice. Journal of Agricultural and Food Chemistry, 68(50), 14748–14757. 10.1021/acs.jafc.0c04748 33264008

[tpj71141-bib-0036] Wang, K. , Guo, G.H. , Bai, S.L. , Ma, J.C. , Zhang, Z. , Xing, Z.Y. et al. (2025) Horizontally acquired CSP genes contribute to wheat adaptation and improvement. Nature Plants, 11(4), 761–774. 10.1038/s41477-025-01952-8 40148598

[tpj71141-bib-0037] Wang, M. , Li, W.Z. , Fang, C. , Xu, F. , Liu, Y.C. , Wang, Z. et al. (2018) Parallel selection on a dormancy gene during domestication of crops from multiple families. Nature Genetics, 50(10), 1435–1441. 10.1038/s41588-018-0229-2 30250128

[tpj71141-bib-0038] Wang, Q. , Lin, Q.B. , Wu, T. , Duan, E.C. , Huang, Y.S. , Yang, C.Y. et al. (2020) OsDOG1L‐3 regulates seed dormancy through the abscisic acid pathway in rice. Plant Science, 298, 110570. 10.1016/j.plantsci.2020.110570 32771171

[tpj71141-bib-0039] Wang, Z. , Zhang, X.F. , Zhou, C. & Cao, X.F. (2025) Control of seed‐to‐seedling transition by an upstream open reading frame in ABSCISIC ACID DEFICIENT2. Proceedings of the National Academy of Sciences of the United States of America, 122(23), e2502155122. 10.1073/pnas.2502155122 PMC12168023 40478869

[tpj71141-bib-0040] Wu, W. , Dong, X.O. , Chen, G.M. , Lin, Z.X. , Chi, W.C. , Tang, W.J. et al. (2024) The elite haplotype OsGATA8‐H coordinates nitrogen uptake and productive tiller formation in rice. Nature Genetics, 56(7), 1516–1526. 10.1038/s41588-024-01795-7 PMC11250373 38872029

[tpj71141-bib-0041] Xiang, D.H. , Tu, H.F. , Yuan, Y. , Yao, Y.L. , Liao, W.W. , Wang, H.J. et al. (2025) A Blast‐resistant NLR gene confers drought resistance by competitively interacting with an E3 ligase to protect phenylalanine ammonia‐lyase in Rice. Advanced Science, 12(39), e02662. 10.1002/advs.202502662 PMC12533315 40686304

[tpj71141-bib-0042] Xu, F. , Tang, J.Y. , Gao, S.P. , Cheng, X. , Du, L. & Chu, C.C. (2019) Control of rice pre‐harvest sprouting by glutaredoxin‐mediated abscisic acid signaling. The Plant Journal, 100(5), 1036–1051. 10.1111/tpj.14501 31436865

[tpj71141-bib-0043] Xu, F. , Tang, J.Y. , Wang, S.X. , Cheng, X. , Wang, H.R. , Ou, S.J. et al. (2022) Antagonistic control of seed dormancy in rice by two bHLH transcription factors. Nature Genetics, 54(12), 1972–1982. 10.1038/s41588-022-01240-7 36471073

[tpj71141-bib-0044] Xu, F. , Yoshida, H. , Chu, C.C. , Matsuoka, M. & Sun, J. (2025) Seed dormancy and germination in rice: molecular regulatory mechanisms and breeding. Molecular Plant, 18(6), 960–977. 10.1016/j.molp.2025.05.010 40420480

[tpj71141-bib-0045] Yang, M.L. , Han, X. , Yang, J.J. , Jiang, Y.J. & Hu, Y.R. (2021) The Arabidopsis circadian clock protein PRR5 interacts with and stimulates ABI5 to modulate abscisic acid signaling during seed germination. The Plant Cell, 33(9), 3022–3041. 10.1093/plcell/koab168 PMC8462813 34152411

[tpj71141-bib-0046] Ye, H. , Feng, J.H. , Zhang, L.H. , Zhang, J.F. , Mispan, M.S. , Cao, Z.Q. et al. (2015) Map‐based cloning of seed Dormancy1‐2 identified a gibberellin synthesis gene regulating the development of endosperm‐imposed dormancy in Rice. Plant Physiology, 169(3), 2152–2165. 10.1104/pp.15.01202 PMC4634095 26373662

[tpj71141-bib-0047] Zeng, R. , Shi, Y.T. , Guo, L. , Fu, D.Y. , Li, M.Z. , Zhang, X.Y. et al. (2025) A natural variant of COOL1 gene enhances cold tolerance for high‐latitude adaptation in maize. Cell, 188(5), 1315–1329. 10.1016/j.cell.2024.12.018 39842436

[tpj71141-bib-0048] Zhang, F.L. , Chi, W.C. , Song, J.W. , Zhu, X.J. , Wang, P. , Chen, Y.Y. et al. (2026) Superior alleles of *SEED‐AGING GENE 9* enhance SEED storability and dormancy in rice. National Science Review, 13(10), nwag248. 10.1093/nsr/nwag248 PMC13248866 42273349

[tpj71141-bib-0049] Zhang, H. , Zhou, J.F. , Kan, Y. , Shan, J.X. , Ye, W.W. , Dong, N.Q. et al. (2022) A genetic module at one locus in rice protects chloroplasts to enhance thermotolerance. Science, 376(6599), 1293–1300. 10.1126/science.abo5721 35709289

[tpj71141-bib-0050] Zhao, B. , Zhang, H. , Chen, T.X. , Ding, L. , Zhang, L.Y. , Ding, X.L. et al. (2022) Sdr4 dominates pre‐harvest sprouting and facilitates adaptation to local climatic condition in Asian cultivated rice. Journal of Integrative Plant Biology, 64(6), 1246–1263. 10.1111/jipb.13266 35442537

[tpj71141-bib-0051] Zhao, X. , Dou, L.R. , Gong, Z.Z. , Wang, X.F. & Mao, T.L. (2019) BES1 hinders abscisic acid insensitive5 and promotes seed germination in Arabidopsis. New Phytologist, 221(2), 908–918. 10.1111/nph.15437 30230549

[tpj71141-bib-0052] Zhao, Y. , Gao, J. , Wang, X.H. , Rashid, M.A.R. , Wu, Z.W. , Ma, Z.Q. et al. (2025) Elite haplotype of STRONG1 enhances rice yield by improving lodging resistance, panicle and plant architecture. Nature Communications, 16(1), 5894. 10.1038/s41467-025-60604-9 PMC12216135 40595499

[tpj71141-bib-0053] Zou, M.J. , Guan, Y.C. , Ren, H.B. , Zhang, F. & Chen, F. (2008) A bZIP transcription factor, OsABI5, is involved in rice fertility and stress tolerance. Plant Molecular Biology, 66(6), 675–683. 10.1007/s11103-008-9298-4 18236009

