# Supplementary material for: SDR3.1 negatively regulates seed dormancy in Poaceae crops
Source: Plant J. 2026 Oct 5;127(6):e71141. doi: 10.1111/tpj.71141 (PMC13639105; doi:10.1111/tpj.71141)
Supplement: Supplementary file 1 [file TPJ-127-0-s001.docx]

*SDR3.1* negatively regulates seed dormancy in Poaceae crops

Naihui Guo^1^, Ruihu An^1^, Mengxue Xu^2^, Yuanyuan Li^1^, Zongliang Ren^1^, Jun Jiang^1^, Bonian Cai^1^, Xinzhu Huang^1^, Shikai Hu^1^, Gaoneng Shao^1^, Guiai Jiao^1^, Lihong Xie^1^, Ling Wang^1^, Fengli Zhao^1^, Yujun Zhu^1^, Shaoqing Tang^1^*, Zhonghua Sheng^1^*, Peisong Hu^1^*

1. State Key Laboratory of Rice Biological Breeding/Key Laboratory of Rice Biology and Breeding, Ministry of Agriculture/China National Rice Improvement Centre/Jiangxi Early-season Rice Research Center/China National Rice Research Institute, Hangzhou, P. R. China

2. Zhejiang top yunnong Technology Co., Ltd, Hangzhou, P. R. China

*Correspondence: Peisong Hu (hupeisong@caas.cn), Shaoqing Tang (sqtang@126.com), Zhonghua Sheng (shengzhonghua666@163.com)

**Figure S1 AI predicts the interaction between SDR3.1 and ABI3. a** SDR3.1 and ABI3 interaction pattern diagram. **b-c** The binding sites of SDR3.1 and ABI3. The red arrow indicates the specific combination position.

**Figure S2 The mutant sites of the *sdr3.1/abi3* double knockout line. a** *SDR3.1* mutant sites of *sdr3.1/abi3* double knockout line; red arrows indicated knockout sites. **b** *ABI3* mutant sites of *sdr3.1/abi3* double knockout line; red arrows indicated knockout sites.

**Figure S3 AI predictions of the interaction between SDR3.1, ABI3, and *Sdr4*. a** ABI3 binds to three sites in the *Sdr4* promoter region. **b** SDR3.1 reduces the binding sites of ABI3 to the *Sdr4* promoter region.

**Figure S4 The mutant sites of the *sdr3.1/sdr4* double knockout line. a** *SDR3.1* mutant sites of *sdr3.1/sdr4* double knockout line; red arrows indicated the knockout sites. **b** *Sdr4* mutant sites of *sdr3.1/sdr4* double knockout line; red arrows indicated the knockout sites.

**Figure S5 SDR3.1 inhibits the expression level of *Sdr4* by ABI5. a** Yeast one-hybrid assay showed that *ABI5* bound to the *Sdr4* promoter. **b** Expression levels of *Sdr4* in *abi5* and ZH11. **c** Schematic of the effectors and reporter used in the transient transactivation assays. **d** SDR3.1 repressed ABI5 to activate *Sdr4* in response to 5 μM ABA.

**Figure S6 The mutant sites of the TM line. a** *SDR3.1* mutant sites of the TM knockout line; red arrows indicated the knockout sites. **b** *ABI3* mutant sites of the TM knockout line; red arrows indicated the knockout sites. **c** *ABI5* mutant sites of the TM knockout line; red arrows indicated the knockout sites.

**Figure S7 PHS of different haplotype combinations.** Five varieties were investigated for each combination, and the survey was conducted four days after rain.

**Figure S8 Homologous analysis of *SDR3.1*. a** Evolutionary tree analysis of *SDR3.1*. **b** Multi-sequence alignment result of *SDR3.1*.

**Figure S9 Homologous analysis of *ABI3*. a** Evolutionary tree analysis of *ABI3*. **b** Multi-sequence alignment result of *ABI3*.

**Figure S10 Y2H verification of SDR3.1 interaction proteins in foxtail millet and maize. a** The Y2H showed that SiABI3 interacted with SiSDR3.1. **b** The Y2H assay showed that ZmABI3 interacted with ZmSDR3.1.

Supplementary Table 1 Germination rate of core parents

| Individual code | Haplotype combination type | Germination rate (%) |
| --- | --- | --- |
| CV1 | m-n | 79.33 |
| CV2 | m-n | 40.67 |
| CV3 | m-n | 26.67 |
| CV4 | m-n | 59.33 |
| CV5 | m-n | 85.33 |
| CV6 | m-n | 84.00 |
| CV7 | m-n | 51.33 |
| CV8 | m-n | 55.33 |
| CV9 | m-n | 60.00 |
| CV10 | m-n | 62.00 |
| CV11 | m-n | 78.00 |
| CV12 | m-n | 66.00 |
| CV13 | m-n | 38.67 |
| CV14 | m-n | 81.33 |
| CV15 | m-n | 8.00 |
| CV16 | m-n | 79.33 |
| CV17 | m-n | 82.67 |
| CV18 | m-n | 84.67 |
| CV19 | m-n | 58.67 |
| CV20 | m-n | 79.33 |
| CV21 | m-n | 30.00 |
| CV22 | m-n | 70.67 |
| CV23 | m-n | 25.33 |
| CV24 | m-n | 50.67 |
| CV25 | m-n | 53.33 |
| CV26 | m-n | 72.67 |
| CV27 | m-n | 48.67 |
| CV28 | m-n | 58.00 |
| CV29 | m-n | 17.33 |
| CV30 | m-n | 15.33 |
| CV31 | m-n | 87.33 |
| CV32 | m-n | 82.67 |
| CV33 | m-n | 84.67 |
| CV34 | m-n | 44.00 |
| CV35 | m-n | 68.67 |
| CV36 | m-n | 85.33 |
| CV37 | m-n | 38.67 |
| CV38 | m-k | 83.33 |
| CV39 | m-k | 83.33 |
| CV40 | m-k | 61.33 |
| CV41 | m-k | 28.67 |
| CV42 | m-k | 60.67 |
| CV43 | h-n | 61.33 |
| CV44 | h-n | 93.33 |
| CV45 | h-n | 28.00 |
| CV46 | h-n | 13.33 |
| CV47 | h-n | 32.67 |
| CV48 | h-n | 58.67 |
| CV49 | h-n | 60.67 |
| CV50 | h-n | 56.00 |
| CV51 | h-n | 36.00 |
| CV52 | h-n | 74.00 |
| CV53 | h-n | 38.00 |
| CV54 | h-n | 91.33 |
| CV55 | h-n | 62.67 |
| CV56 | h-n | 76.67 |
| CV57 | h-n | 36.67 |
| CV58 | h-n | 86.00 |
| CV59 | h-n | 94.67 |
| CV60 | h-n | 64.00 |
| CV61 | h-n | 80.00 |
| CV62 | h-n | 18.00 |
| CV63 | h-n | 82.00 |
| CV64 | h-n | 80.00 |
| CV65 | h-n | 92.00 |
| CV66 | h-n | 70.00 |
| CV67 | h-n | 91.33 |
| CV68 | h-n | 82.00 |
| CV69 | h-n | 13.33 |
| CV70 | h-n | 64.67 |
| CV71 | h-n | 44.67 |
| CV72 | h-n | 25.33 |
| CV73 | h-n | 94.00 |
| CV74 | h-n | 71.33 |
| CV75 | h-n | 62.67 |
| CV76 | h-n | 80.00 |
| CV77 | h-n | 88.00 |
| CV78 | h-n | 70.67 |
| CV79 | h-n | 86.00 |
| CV80 | h-n | 76.00 |
| CV81 | h-n | 80.00 |
| CV82 | h-n | 62.67 |
| CV83 | h-n | 33.33 |
| CV84 | h-n | 82.67 |
| CV85 | h-n | 76.00 |
| CV86 | h-n | 69.33 |
| CV87 | h-n | 54.00 |
